# Parallel exploratory and confirmatory factor analysis of the Hungarian Fear of COVID-19 Scale in a large general population sample: a psychometric and dimensionality evaluation

**DOI:** 10.1186/s12889-022-13789-3

**Published:** 2022-07-28

**Authors:** Péter György Balázs, Ariel Mitev, Valentin Brodszky

**Affiliations:** 1 Corvinus University of Budapest, Budapest, Hungary; 2 Corvinus University of Budapest, Doctoral School of Business and Management, Budapest, Hungary

**Keywords:** COVID-19, Fear, Confirmatory factor analysis, Hungarian FCV-19S, Psychometrics

## Abstract

**Background:**

This study aims to confirm validity and reliability of the Hungarian version of Fear of COVID-19 Scale (FCV-19S) and evaluate its dimensional structure.

**Methods:**

Cross-sectional survey was carried out in 2021 among Hungarian general population. In addition to classical test theory methods, construct dimensionality of FCV-19S was assessed using EFA with principal axis factoring method and CFA with diagonally-weighted least squares estimation. Fear score was compared in age, gender, educational level, vaccination and infection subgroups.

**Results:**

Significant differences in FCV-19S mean scores were observed between three subgroups (age, gender, vaccination). Items showed good internal consistency (Cronbach α = 0.88). EFA identified two latent factors (eig = 4.2 and 1.02), though parallel analysis supports the one-factor model. The two-dimensional structure was confirmed by CFA, items 3,4,6,7 correlated with Factor 1 (physiological fear), items 1,2,5 with Factor 2 (emotional fear).

**Conclusion:**

The Hungarian version of FCV-19S seems valid and reliable. The EFA identified two-latent factors (emotional and physiological fear), that was confirmed by CFA. The two-factor structure had better model fit, though its’ acceptance is limited.

**Supplementary Information:**

The online version contains supplementary material available at 10.1186/s12889-022-13789-3.

## Introduction

The COVID-19 pandemic raised rapid public health emergency globally from its appearance in December 2019. Hungary imposed containment phase in 2020 March, to prevent steep shift in epidemiological curve. The government implemented several pre-emptive policies such as domestic/ international travel restrictions, shuttering business activity, distance learning in public & university education. Besides the economic backlash, sudden lockdown multiplied with the risks posed by an unknown infectious disease resulted in wide-range of impacts (such as anxiety, stress, problems in social and emotional functioning) on health-related quality of life (HRQoL) of the population [1, 2]. Mass vaccination started in 2021 mid-January, shortly followed by the third wave of the pandemic (March-June 2021), bringing sanctuary regulations back (distance learning in public education, austere lockdown, mandatory mask-wearing in/outside).

Similarly, to other countries, Hungary started to investigate several related aspects of the pandemic, including fear, anxiety, stigmatization and worries [3–5]. Several studies address fear as the most contagious factor of epidemics [6–13]. The uniqueness of the COVID-19 stressed that instead of utilizing generic psychological scales, developing a new disease-specific Fear of COVID-19 Scale (FCV-19S) would be adequate.

The first survey introducing the FCV-19S that has been used to measure fear associated with COVID-19 pandemic was published in March 2020 [14]. Currently it has been validated in at least 40 countries and translated to more than 20 languages (see supplementary material). The Hungarian version of FCV-19S has been validated in a convenient sample of university students and lecturers in 2021 [15].

Despite of many existing scales measuring fear and anxiety of individuals, the crucial role of fear in response to global pandemic outbreak generated demand in research of psychology and related disciplines towards the FCV-19 Scale. It is directly designed to measure fear of COVID-19 and able to identify vulnerable population groups. Pandemic caused fear of dying, getting infected, unable to work may aggregate in stress/anxiety that negatively impacts people’s health [16–19]. Health policy interventions need appropriate information of COVID-19 generated fear, that is measured by a pandemic-specific instrument [14, 20].

The rapid spread of FCV-19S generated information in conclusions of good internal consistency, acceptable construct, convergent & concurrent validity, with satisfactory psychometric properties of the scale [6, 21–24]. The psychometric tests of FCV-19S suggest that it is applicable in all genders and age-groups [25]. However, there is a debate in the number of underlying latent factors [26–34].

Methods of classical test theory (CTT) (e.g. item-correlations, Cronbach’s α) and item response theory (IRT), also referred as modern test theory (e.g. parameter logistic modelling, Rasch analysis) may all be used to assess the validity and reliability of a new measurement tool. Dimensionality of constructs is often explored by exploratory/confirmatory factor analysis (EFA and/or CFA). Besides CTT, IRT and factor analysis elements, more researches expanded the instrument evaluation with structural equation modelling (SEM) [35, 36] or Rasch analysis [14, 25].

Initially the FCV-19S was designed to capture one-dimension of fear. Several studies confirmed the one-dimensional structure of the scale using either modified [20, 37–40] or the initial CFA model [41]. When designing a new construct to measure a physiological/psychological dimension (e.g. pain or fear), preferably the item numbers should be balanced related to each dimension, particularly when more than one latent factor may be present. So far, several studies have approved the one-dimensional structure of the FCV-19S [15, 42, 43], although other evaluations reported valid and reliable two-dimensional factor structures identifying both emotional and physiological factors of fear [31, 44]. The two-factor models were inconclusive, divergent factor structures appeared [29, 40, 41]. To date only few studies cross-compared directly the one and two-factor models to examine a potential two factor structure of FCV-19S [27, 34, 45].

### Objective

Compared to the huge number of construct dimensionality evaluation studies using one-factor structure [20, 24, 25, 46, 47], only few completed two-factor models [22, 27, 29, 31, 48], even fewer reported the results of EFA and CFA cross-comparison. This study aims to compare the one and two-factor structure by EFA and CFA dimensionality assessment and analyse the psychometric properties of the FCV-19S.

## Methods

### Data collection method

A large-scale cross-sectional survey was carried out between May 25^th^ – June 08^th^ 2021. Similarly to previous FCV-19S validation studies, an online self-completed questionnaire has been developed. A professional survey company collected the data, using their online panel database. The targeted sample size was *N* = 2,000 of Hungarian adult general population, no data was collected from dropouts.

### Fear of COVID-19 Scale

The FCV-19S questionnaire consists of seven-items scored on a five-point Likert scale, with maximum overall score of 35 (indicating the highest level of fear) [14]. Respondents are questioned about the extent of being afraid of coronavirus (anxiousness, losing life, uncomfortable thoughts) and the related physiological manifestations of fear (palpitating heart, clammy hands, losing sleep). Seven closed questions, each represented by one statement, rated on a 1–5 point balanced scale (‘1’ indicating strong disagreement) measured respondents fear related to coronavirus.

### Translation

The permission to translate and utilize FCV-19S scale was obtained from the developer team of the questionnaire. To adapt it, forward–backward translation was applied supervised by two health professionals and harmonized by group discussion of more researchers. The original translation was altered in item 1 (most afraid of coronavirus) and item 5 (nervous and anxious when watching news and stories about coronavirus): to (1) being very afraid and (5) see news in social media, in order to comply cultural and semantical embedding. “Coronavirus” appellation was changed to “COVID-19”. The final version has been back-translated to English by a third independent native speaker, the consensus version needed no changes. (See the Hungarian version in Supplementary material).

### Statistical analysis

Descriptive statistics including means and central tendency measures were used to explore sample population and item characteristics (standard deviation, variance, skewness, kurtosis, floor effect, ceiling effect). Reliability of the FCV-19 Scale was examined by CTT, measuring items correlation (Pearson’s r and corrected item-total correlation) and internal consistency (Cronbach alpha). Inflation in mean estimates was assessed through ceiling and floor effect with 95% confidence interval consideration. Interpretation values for FCV-19S construct properties were set as Pearson correlation coefficient for inter item consistency > 0.3; Cronbach alpha for internal consistency > 0.7 and corrected item–total correlation coefficient with a value of no less than 0.5 [49, 50].

Dimensionality information of the 7-item construct was revealed by conducting a separate one- and two-factor model exploratory factor analysis, followed by confirmatory factor analysis, using principal axis factoring extraction for EFA. Diagonally-weighted least square (DWLS) estimation was used for CFA, due to better fit to ordinal data [51, 52]. The sample was randomly distributed into two subsamples (50–50%) to cross-validate EFA & CFA measures [53]. Communalities, factor loadings, standardized estimates, regression weights and squared multiple correlations (SMC) were reported. Factor loadings were interpreted as acceptable if ≥ 0.3, practically significant if ≥ 0.5 and indicative of a well-defined structure if ≥ 0.7 [54]. Rotation method is decided based on factors correlation, distribution is considered oblige in component correlation is above 0.4. Absolute model fit measures in terms of root mean square error of approximation (RMSEA), standardized mean root square residual (SRMR), Chi square and degree of freedom were compared, such as incremental model fit indices of Tucker–Lewis index (TLI) and comparative fit index (CFI). Common threshold values were used both for RMSEA ≤ 0.08 and SRMR ≤ 0.06 [55] ;  > 0.95 for TLI and CFI [56].

Nonparametric tests (Kruskal–Wallis and Mann–Whitney) were used to compare FCV-19S mean scores of subgroups to demonstrate construct validity. Convergent validity between FCV-19S score and health status measurement tool scores: the Hungarian value set based EQ-5D-5L utility index [57, 58], seven item General Anxiety Disorder (GAD-7) [59] and nine item Patient Health Questionnaire (PHQ-9) [60] was explored using Spearman’s correlation. Statistical analysis was carried out using IBM SPSS (Version 25.0. Armonk, NY: IBM Corp), factor analysis was performed using R (v4.1.2; R Core Team 2021; levaan 0.6–11 package).

## Results

### Population characteristics

Overall, *N* = 2,421 started the questionnaire, the final sample contains *N* = 2000 complete responses of the Hungarian adult general population (response rate: 82.6%). The mean age was 49.1 (SD = 15.3), majority of the sample was female (*n* = 1244; 62.2%). Education level of respondents in primary, secondary and tertiary school distributed as follows: 21.8%, 45.0%, 33.2%. Majority of the sample population has been vaccinated (*n* = 67.3%), and 18.5% (*n* = 370) already encountered the infection. At the time of data collection altogether 5.1 million people (53%) in Hungary were vaccinated at least once [61] (Table 1).

**Fig. 1:**
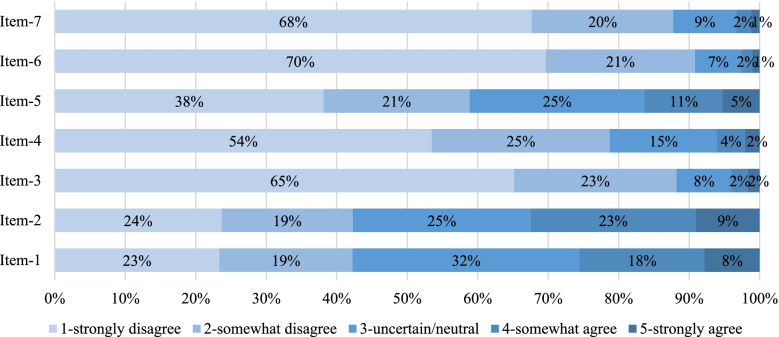
Distribution of responses among items

Total average fear score out of the possible maximum of 35 on FCV-19S was 13.9 (SD = 5.5). Central tendency measures suggest that all people agreed the most with the statements 1, 2 and 5 indicating the highest impact of unpleasant thinking, being afraid, and seeing news of COVID-19 on fear level. Statement 1 (*n* = 26%), 2 (*n* = 32%), 5 (*n* = 16%) had the highest number of agreements while the same items (1,2 and 5) indicated the greatest uncertainty (*n* = 32%, 25%, 25%). Response distribution among items, showed gravitation towards high disagreement in questions 3 (*n* = 83%), 4 (*n* = 79%), 6 (*n* = 91%) and 7 (*n* = 88%) (Fig. 1).

Nonparametric tests revealed significant differences in FCV-19S scores among separate age (*p* < 0.01), gender (*p* < 0.001) and vaccination groups (*p* < 0.001). Older individuals (aged > 55+), women and vaccinated had higher mean scores, demonstrating elevated fear due COVID-19.

Health status measure scores all correlated significantly (*p* < 0.01) on slight to moderate level with the FCV-19S: GAD-7 (rho = 0.373); PHQ-9 (rho = 0.309); EQ-5D-5L index (rho = -0.235) demonstrating convincing convergent validity (Tables 2 and 3).

### Consistency measures

The total internal consistency of the COVID-19 fear questionnaire was good (Cronbach α = 0.88), all items indicated proper internal consistency, corrected item-total correlations of items range between 0.62–0.75. All seven items were significantly correlating (*p* < 0.001) on a moderate to high level (*r* = 0.357–812), expressing acceptable instrument validity and enabling factor analysis (Kaiser–Meyer–Olkin measure = 0.864; *p* < 0.001).

### Factor analysis outcomes

Results of one-factor EFA show that items 4, 6 and 7 have high level of common variance (h^2^ = 0.69; 0.60; 0.68). Total variance explained by the one-factor model was 60.8%, with component’s eigenvalue = 4.26 meaning that the factor explains the same amount of variance than 4.2 items. Oblimin rotation with Kaiser normalization was applied in case of the two-factor model: factors were correlating r = 0.61 (coefficients smaller than 0.30 were supressed in the pattern matrix). All items have moderate/high common variance (h^2^ = 0.54–0.80). Factor loading shows that questions 1,2,5 likely belong to Factor 1, named as “emotional fear” based on item correlation strength. Questions 3,4,6,7 are strongly related to Factor 2, named as “physiological fear”. Factor retaining was decided by parallel analysis, the random data eigenvalue score was much bigger (eig = 1.069), than the second factors eigenvalue (1.022) of the sample (Table 4).

**Table 1:** Sample characteristics and mean FCV-19S scores

Variable		n	%	FCV-19S score (SD)	*p* value*
Total		2000	100	13.9 (5.5)	
Age group	18–34	437	21.9	13.5 (5.8)	< *0.001*
	35–54	763	38.1	13.7 (5.7)	
	55 <	800	40.0	14.3 (5.2)	
Gender	male	756	37.8	13.1 (5.3)	< *0.001*
	female	1244	62.2	14.4 (5.7)	
Educational level	primary	435	21.8	14.7 (6.6)	= 0.166
	secondary	900	45.0	13.8 (5.4)	
	tertiary	665	33.2	13.5 (4.9)	
Vaccinated	yes	1347	67.3	14.2 (5.3)	< *0.001*
	no	653	32.7	13.1 (6.0)	
Infected	yes	370	18.5	13.8 (5.7)	= 0.551
	no	1630	81.5	13.9 (5.5)	

^*^Known-group validity was assessed by the appropriate Kruskal–Wallis H or Mann–Whitney U non-parametric test (significant if *p* < 0.05)

**Table 2:** Descriptive statistics of item responses

Descriptive statistics of FCV-19S items in total sample								
Items	n	mean	SD	skewness	kurtosis	floor %	ceiling %	Item variance
Item #1	2000	2.68	1.23	0.13	-0.93	23.4	7.8	1.51
Item #2	2000	2.76	1.29	0.05	-1.15	23.7	9.0	1.67
Item #3	2000	1.52	0.86	1.92	3.74	65.2	1.7	0.74
Item #4	2000	1.76	0.99	1.27	1.05	53.5	2.1	0.98
Item #5	2000	2.25	1.22	0.59	-0.70	38.2	5.3	1.48
Item #6	2000	1.43	0.77	2.11	4.89	69.7	1.0	0.59
Item #7	2000	1.49	0.84	1.89	3.50	67.7	1.2	0.70
Descriptive statistics of items in vaccinated group								
Item #1	1347	2.85	1.19	-0.04	-0.87	17.4	8.4	1.43
Item #2	1347	2.84	1.25	-0.04	-1.07	19.5	8.3	1.55
Item #3	1347	1.54	0.83	1.73	2.95	62.8	1.0	0.70
Item #4	1347	1.8	0.98	1.15	0.76	50.3	1.8	0.96
Item #5	1347	2.29	1.19	0.47	-0.8	35.3	4.3	1.42
Item #6	1347	1.43	0.73	1.89	3.71	68.7	0.4	0.54
Item #7	1347	1.49	0.82	1.77	2.85	67.1	0.7	0.67
Descriptive statistics of items in non-vaccinated group								
Item #1	653	2.31	1.22	0.53	-0.63	35.5	6.6	1.49
Item #2	653	2.59	1.37	0.26	-1.20	32.2	10.6	1.88
Item #3	653	1.49	0.92	2.24	4.90	70.1	2.9	0.85
Item #4	653	1.68	1.00	1.52	1.76	60.2	2.8	1.01
Item #5	653	2.15	1.27	0.81	-0.45	44.0	7.2	1.62
Item #6	653	1.43	0.84	2.38	6.03	71.7	2.1	0.71
Item #7	653	1.49	0.87	2.11	4.58	68.9	2.1	0.75

**Table 3:** Correlation of the seven items

Pearson’s correlation of FCV-19S items								Corrected item-total correlation	Cronbach alpha if item deleted
Items	Item #1	Item #2	Item #3	Item #4	Item #5	Item #6	Item #7		
Item #1	1							0.617	0.866
Item #2	0.601	1						0.626	0.866
Item #3	0.403	0.402	1					0.663	0.859
Item #4	0.544	0.471	0.652	1				0.749	0.847
Item #5	0.499	0.611	0.494	0.589	1			0.699	0.853
Item #6	0.366	0.357	0.625	0.618	0.486	1		0.666	0.861
Item #7	0.418	0.405	0.634	0.646	0.537	0.812	1	0.711	0.855

**Table 4:** One and two-factor EFA results

EFA model	One-factor model		Two-factor model		
Items (question)	Communality (h^2^)	Factor matrix (1 factor loading)	Communality (h^2^)	Factor matrix	
				1-physiological factor	2-emotional factor
Item-1	0.389	0.575	0.536	-	0.698
Item-2	0.380	0.558	0.680		0.874
Item-5	0.540	0.691	0.593		0.587
Item-3	0.560	0.760	0.563	0.640	-
Item-4	0.691	0.819	0.666	0.605	
Item-6	0.595	0.828	0.803	0.967	
Item-7	0.667	0.857	0.787	0.890	

The one-factor model CFA estimated one-unit change in standard deviation of items between β = 0.65–0.81, modest in case of item 1, highest in item 4. Unstandardized regression estimate showed that elevation in fear score increases item scores (unstandardized β = 0.63–1.14). Predicted variance of items ranged between R^2^ = 42.8–65.3%. No covariances were drawn between items, the baseline model showed acceptable model fit both in one- and two-factor structure (Table 5).

**Table 5:** Results of one and two-factor CFA

**One-factor CFA model**				
*Item*	*Latent factor*	*estimate (stand. reg. weight)*	*regression weight (SE)*	*squared loading (SMC)*
#1	Fear	0.654	1.00 (-)	0.428
#2		0.670	1.08 (0.05)	0.449
#3		0.684	0.74 (0.04)	0.468
#4		0.808	1.01 (0.05)	0.653
#5		0.759	1.14 (0.06)	0.576
#6		0.668	0.63 (0.04)	0.447
#7		0.727	0.74 (0.04)	0.529
**One-factor model fit**				
*Statistical measure*	*Test indices*	*Test Results*	*Test standard (unit value)*	*Model fit*
*Absolute fit*	RMSEA	0.075	< 0.08	excellent
	Chi square/df	93.3 (14)	< 5.0	not optimal
	Chi square p	< 0.001	> 0.05	not optimal
	SRMR	0.091	< 0.06	not optimal
*Incremental fit*	TLI	0.974	> 0.95	good
	CFI	0.962	> 0.95	good
**Two-factor CFA model**				
*Item*	*Latent factor*	*estimate (stand. reg. weight)*	*regression weight*	*squared loading (SMC)*
#1	1. factor: emotional fear	0.705	1.00 (-)	0.498
#2		0.735	1.10 (0.05)	0.540
#5		0.805	1.13 (0.06)	0.649
#3	2. factor: physiological fear	0.755	1.00 (-)	0.571
#4		0.877	1.34 (0.08)	0.769
#6		0.743	0.86 (0.05)	0.552
#7		0.806	1.00 (0.06)	0.649
**Two-factor model fit**				
*Statistical measure*	*Test indices*	*Test Results*	*Test standard (unit value)*	*Model fit*
*Absolute fit*	RMSEA	0.044	< 0.08	excellent
	Chi square/df	38.3 (13)	< 5.0	optimal
	Chi square p	< 0.001	> 0.05	not optimal
	SRMR	0.057	< 0.06	good
*Incremental fit*	TLI	0.992	> 0.95	excellent
	CFI	0.987	> 0.95	excellent

The two-factor model had better model fit in terms of RMSEA, Chi-square/df, SRMR, TLI and CFI. The one-unit change impact of “emotional fear” factor on items 1, 2, 5 (β = 0.71; 0.74; 0.81) was similar to the impact of “physiological fear” factor on items 3, 4, 6, 7 (β = 0.76; 0.88; 0.74; 0.81). Predictors of items explained R^2^ = 49.8–76.9% of items variance. In comparison to the one-factor model, the error variance of items in the two-factor model ranged on larger scale (34.7–57.2 vs 23.1–50.2%). Fear elevated in items 2 & 5 (uncomfortable to think on coronavirus-19 & becoming nervous) due emotional fear factor (unstandardized β = 1.10 & 1.13). Fear was elevating in items 4,6,7 (losing life; can’t sleep; heart palpitates) due physiological fear factor (unstandardized β = 1.34; 0.86; 1.00). Emotional and physiological factors showed linear covariation (0.43) and significant high level of correlation (*r* = 0.76) (Table 5).

## Discussion

A large cross-sectional study among general population to validate the Hungarian seven item FCV-19S was done in 2021 May. Psychometric properties were analysed by descriptive statistics, methods of classical test theory were used to measure validity and reliability. Exploratory and confirmatory factor analysis was conducted to compare one and two-factor structure of the construct.

Differences in FCV-19S mean scores were highlighted between different age groups (18–34: 13.5; 35–54: 13.7; 55 ≤ : 14.3), male (13.1) and female (14.4), vaccinated (14.2) and non-vaccinated (13.1) population groups. Correlation between anxiety (GAD-7), depression (PHQ-9) and FCV-19S was moderate, while EQ-5D utility and fear score weakly correlated. Item descriptive measures suggest that people agreed the most with questions 1 (afraid of COVID-19), 2 (unpleasant thinking) and 5 (seeing news in social media). Vaccinated group indicated higher fear in items 1–5 and equalled in items 6–7 compared to non-vaccinated group. Items 3 (clammy hands), 4 (losing life), 6 (cannot sleep), 7 (palpitating heart) received the biggest proportion of disagreement responses.

The Hungarian version of the FCV-19S has good level of internal consistency (Cronbach’s α = 0.88), and high corrected item-total correlation (*r* = 0.666–0.749). Dimensionality assessment by one and two-factor exploratory factor analysis, suggests that observing two latent factors is possible, also resulted a better model fit, whereas the eigenvalue of the second latent factor was arguably low (eig = 1.02). The randomly generated correlation matrices of parallel analysis suggest minimum mean eigenvalue of no less than 1.07 for the second factor, supporting the one factor structure. Similarly, a large cross-country comparative analysis further supported the unidimensional structure of the construct [62].

All previously published two-factor analyses showed good model fits, while the item-factor structures turned out different [27, 29, 31, 63]. Almost every two-factor analyses identified items 1,2,4,5 as “emotional fear” factor and items 3,6,7 as “physiological component” of fear, respectively [27, 31, 34, 44, 48, 63]. In one study [29], items 1,2,4 represented “cognitive fear” factor and 3,5,6,7 the “somatic fear” factor. Our two-factor structure is unique: physiological fear factor consists of items 3,4,6,7 and emotional fear of items 1,2,5. The weakest element in our two-factor structure, shown by the pattern matrix was item 4 (better correlating with the physiological factor: 0.61 vs 0.29). Nevertheless, item 4 potentially belongs to emotional fear dimension, albeit CFA model fit results were better, when classifying it to physiological fear dimension. The results of factor structures in many studies echoes strong relationship (covariance) in item 1–2 and item 6–7, simultaneously a gap between these pairs [26, 37, 38, 40]. Two-latent factors practically imply that COVID-19 fear incorporate emotional and physiological fear, that is heterogeneous among people, thus both factors should be separately measured to differentiate between observational and latent factors. Although, standardization of a composite fear score would be extremely difficult, mostly due to the divergences in factor structures. This inconsistency of the dimensional structure of FCV-19S further supports the acceptance of the one-dimensional approach.

Contemporary study introduced the validation of the Hungarian version of the FCV-19 Scale, showing congruent results to our study, though research groups worked separately [15]. Although, minor differences in translation regarding synonyms appeared, literally the sentences were corresponding. The two study samples (convenient vs general population) and data collection time (2021 January: mid of second wave vs 2021 May: ending of the third wave) remarkably differed. Conclusions of the two studies on excellent construct validity and reliability were similar. Dimensionality assessed only by one-factor CFA, showed dissimilar, rather poor model fit (RMSEA: 0.16 vs 0.08 and CFI: 0.84 vs 0.97), compared to our results, but factor loadings were kindred (0.47–0.84 vs 0.65–0.81).

This study aimed to evaluate the (1) psychometric properties, (2) validity and reliability and (3) dimensionality of the Hungarian version of Fear of COVID-19 Scale, although a primary limitation is posed by the timing of the data collection. Salient divergences in COVID-19 fear could have been between the contagion peaks of the pandemic and access to vaccines both within the country and between countries. Second limitation of this study is the online data collection and convenient sampling among respondents of panel database. Considering the second factors eigenvalue (1.02), EFA and parallel analysis suggests that researchers have to be cautious when concluding the results of the two-factor model. Item 4 (losing life), unlike in other structures was stronger related to physiological than to emotional fear factor.

## Conclusion

Huge number of psychometric validation and factor analysis studies followed the original FCV-19S validation study [14]. Our results are consistent with the previous findings [20, 42, 43, 64], emphasising that FCV-19S has reliable and valid measurement properties.

We used one- and two-factor EFA along with parallel analysis to evaluate the dimensionality of the construct. Two latent factors (emotional and physiological fear) were confirmed by CFA, moreover the two-factor structure showed better model fit, though its’ generalization faces limitations. Although the FCV-19S original factor structure was one-dimensional, our findings suggest a two-factor structure.

## Supplementary Information

**Additional file 1.**

**Additional file 2.**
