# Supplementary material for: Parallel exploratory and confirmatory factor analysis of the Hungarian Fear of COVID-19 Scale in a large general population sample: a psychometric and dimensionality evaluation
Source: BMC Public Health. 2022 Jul 28;22:1438. doi: 10.1186/s12889-022-13789-3 (PMC9333073; doi:10.1186/s12889-022-13789-3)
Supplement: Supplementary file 1 — Additional file 1. [file 12889_2022_13789_MOESM1_ESM.docx]

The first survey introducing the Fear of Covid-19 Scale that has been used to measure fear associated with COVID-19 pandemic was published in March 2020 (1). Currently it has been validated in at least 40 countries: Argentina (2), Bangladesh (3), Brazil (4), China (5), Colombia (6), Cuba (7), Dominican Republic (8), Egypt (9), Ethiopia (10), France (11), Greece (12), Hungary (13), Jordan (14), India (15), Indonesia (16), Iran (1), Italy (17), Israel (18), Japan (19, 20), Malaysia (21), Mexico (22), Mozambique (23), New Zealand (24), Nigeria (25), Norway (26), Pakistan (27), Paraguay (28), Peru (29), Poland (30), Portugal (31), Romania (32), Russia (33), Saudi Arabia (34), South Africa (35), South Korea (36),Spain (8), Taiwan (37), Turkey (38), United States (39), United Kingdom (40), Vietnam (41)) and translated to more than 20 languages.

| **FCV-19 questionnaire validations** | | | |
| --- | --- | --- | --- |
| **Country** | **Language** | **Autor(s)** | **year** |
| Argentina | Spanish | Caycho-Rodríguez et al. | 2020 |
| Bangladesh | Bangla | Sakib et al. | 2020 |
| Brazil | Portuguese | Cavalheiro et al. | 2020 |
| China | Chinese (mandarin) | Chi et al. | 2021 |
| Colombia | Spanish | Merkado-Lara et al. | 2021 |
| Cuba | Spanish | Bonche-Perez et al. | 2020 |
| Dominican Republic | Spanish | Piqueras et al. | 2021 |
| Egypt | Arabic | Fawzy El-Bardan et al. | 2021 |
| Ethiopia | Amharic | Elemo et al. | 2020 |
| France | French | Mailliez et al. | 2021 |
| Greece | Greek | Nikopoulou et al. | 2020 |
| Hungary | Hungarian | Stankovic et al. | 2021 |
| Jordan | Arabic | Al-Shannaq et al. | 2021 |
| India | English | Bellamkonda et al. | 2021 |
| Indonesia | Indonesian | Nazari et al. | 2021 |
| Iran | Persian | Ahorsu et al. | 2020 |
| Italy | Italian | Soraci et al. | 2020 |
| Israel | Hebrew | Tzur Bitan et al. | 2020 |
| Japan | Japanese | Masuyama et al. & Wakashima et al. | 2020 |
| Malaysia | Malay | Pang et al. | 2020 |
| Mexico | Spanish | García-Reyna et al. | 2020 |
| Mozambique | Portuguese | Giordani et al. | 2021 |
| New Zeland | English | Winter et al. | 2020 |
| Nigeria | English | Chia et al. | 2021 |
| Norway | Norwegian | Iversen et al. | 2021 |
| Pakistan | Urdu | Mahmood et al. | 2020 |
| Paraguay | Spanish | Barrios et al. | 2021 |
| Peru | Spanish | Huarcaya-Victoria et al. | 2020 |
| Poland | Polish | Pilch et al. | 2021 |
| Portugal | Portuguese | Magano et al. | 2021 |
| Romania | Romanian | Stănculescu et al. | 2021 |
| Russia | Russian | Reznik et al. | 2020 |
| Saudi Arabia | Arabic | Alyami et al. | 2020 |
| South Africa | not reported | Pretorius et al. | 2021 |
| South Korea | Korean | Han et al. | 2021 |
| Spain | Spanish | Piqueras et al. | 2021 |
| Taiwan | not reported | Chang et al. | 2020 |
| Turkey | Turkish | Satici et al. | 2020 |
| United States | English | Perz et al. | 2020 |
| United Kingdom | English | Harper et al. | 2020 |
| Vietnam | Vietnamese | Nguyen et al. | 2020 |

1. Ahorsu DK, Lin CY, Imani V, Saffari M, Griffiths MD, Pakpour AH. The Fear of COVID-19 Scale: Development and Initial Validation. International journal of mental health and addiction. 2020; doi:10.1007/s11469-020-00270-8

2. Caycho-Rodríguez T, Vilca LW, Cervigni M, Gallegos M, Martino P, Portillo N, et al. Fear of COVID-19 scale: Validity, reliability and factorial invariance in Argentina's general population. Death studies. 2020; doi:10.1080/07481187.2020.1836071

3. Sakib N, Bhuiyan A, Hossain S, Al Mamun F, Hosen I, Abdullah AH, et al. Psychometric Validation of the Bangla Fear of COVID-19 Scale: Confirmatory Factor Analysis and Rasch Analysis. International journal of mental health and addiction. 2020; doi:10.1007/s11469-020-00289-x

4. Cavalheiro FRS, Sticca MG. Adaptation and Validation of the Brazilian Version of the Fear of COVID-19 Scale. International journal of mental health and addiction. 2020; doi:10.1007/s11469-020-00415-9

5. Chi X, Chen S, Chen Y, Chen D, Yu Q, Guo T, et al. Psychometric Evaluation of the Fear of COVID-19 Scale Among Chinese Population. International journal of mental health and addiction. 2021; doi:10.1007/s11469-020-00441-7

6. Mercado-Lara MF, Campo-Arias A, Monterrosa-Castro Á. Validity and Reliability of the Spanish Version of Fear of COVID-19 Scale in Colombian Physicians. International journal of mental health and addiction. 2021; doi:10.1007/s11469-020-00430-w

7. Broche-Pérez Y, Fernández-Fleites Z, Jiménez-Puig E, Fernández-Castillo E, Rodríguez-Martin BC. Gender and Fear of COVID-19 in a Cuban Population Sample. International journal of mental health and addiction. 2020; doi:10.1007/s11469-020-00343-8

8. Piqueras JA, Gomez-Gomez M, Marzo JC, Gomez-Mir P, Falco R, Valenzuela B, et al. Validation of the Spanish Version of Fear of COVID-19 Scale: its Association with Acute Stress and Coping its Association with Acute Stress and Coping. International journal of mental health and addiction. 2021; doi:10.1007/s11469-021-00615-x

9. Fawzy El-Bardan M, Lathabhavan R. Fear of COVID-19 scale: Psychometric properties, reliability and validity in Egyptian population. Diabetes & metabolic syndrome. 2021; doi:10.1016/j.dsx.2021.05.026

10. Elemo AS, Satici SA, Griffiths MD. The Fear of COVID-19 Scale: Psychometric Properties of the Ethiopian Amharic Version. International journal of mental health and addiction. 2020; doi:10.1007/s11469-020-00448-0

11. Mailliez M, Griffiths MD, Carre A. Validation of the French Version of the Fear of COVID-19 Scale and Its Associations with Depression, Anxiety, and Differential Emotions. International journal of mental health and addiction. 2021; doi:10.1007/s11469-021-00499-x

12. Nikopoulou VA, Holeva V, Parlapani E, Karamouzi P, Voitsidis P, Porfyri GN, et al. Mental Health Screening for COVID-19: a Proposed Cutoff Score for the Greek Version of the Fear of COVID-19 Scale (FCV-19S). International journal of mental health and addiction. 2020; doi:10.1007/s11469-020-00414-w

13. Stankovic M, Papp L, Nyúl B, Ivánkovits L, Pető Z, Töreki A. Adaptation and psychometric evaluation of Hungarian version of the Fear of COVID-19 Scale. PloS one. 2021; doi:10.1371/journal.pone.0261745

14. Al-Shannaq Y, Mohammad AA, Khader Y. Psychometric Properties of the Arabic Version of the Fear of COVID-19 Scale (FCV-19S) Among Jordanian Adults. International journal of mental health and addiction. 2021; doi:10.1007/s11469-021-00574-3

15. Bellamkonda N, Pattusamy M. Validation of Fear of COVID-19 Scale in India: Classical Test Theory and Item Response Theory Approach. International journal of mental health and addiction. 2021; doi:10.1007/s11469-021-00521-2

16. Nazari N, Safitri S, Usak M, Arabmarkadeh A, Griffiths MD. Psychometric Validation of the Indonesian Version of the Fear of COVID-19 Scale: Personality Traits Predict the Fear of COVID-19. International journal of mental health and addiction. 2021; doi:10.1007/s11469-021-00593-0

17. Soraci P, Ferrari A, Abbiati FA, Del Fante E, De Pace R, Urso A, et al. Validation and Psychometric Evaluation of the Italian Version of the Fear of COVID-19 Scale. International journal of mental health and addiction. 2020; doi:10.1007/s11469-020-00277-1

18. Tzur Bitan D, Grossman-Giron A, Bloch Y, Mayer Y, Shiffman N, Mendlovic S. Fear of COVID-19 scale: Psychometric characteristics, reliability and validity in the Israeli population. Psychiatry research. 2020; doi:10.1016/j.psychres.2020.113100

19. Masuyama A, Shinkawa H, Kubo T. Validation and Psychometric Properties of the Japanese Version of the Fear of COVID-19 Scale Among Adolescents. International journal of mental health and addiction. 2020; doi:10.1007/s11469-020-00368-z

20. Wakashima K, Asai K, Kobayashi D, Koiwa K, Kamoshida S, Sakuraba M. The Japanese version of the Fear of COVID-19 scale: Reliability, validity, and relation to coping behavior. PloS one. 2020; doi:10.1371/journal.pone.0241958

21. Pang NTP, Kamu A, Hambali NLB, Mun HC, Kassim MA, Mohamed NH, et al. Malay Version of the Fear of COVID-19 Scale: Validity and Reliability. International journal of mental health and addiction. 2020; doi:10.1007/s11469-020-00355-4

22. García-Reyna B, Castillo-García GD, Barbosa-Camacho FJ, Cervantes-Cardona GA, Cervantes-Pérez E, Torres-Mendoza BM, et al. Fear of COVID-19 Scale for Hospital Staff in Regional Hospitals in Mexico: a Brief Report. International journal of mental health and addiction. 2020; doi:10.1007/s11469-020-00413-x

23. Giordani RCF, Giolo SR, Muhl C, Estavela AJ, Mabuie Gove JI. Validation of the FCV-19 Scale and Assessment of Fear of COVID-19 in the Population of Mozambique, East Africa. Psychology research and behavior management. 2021; doi:10.2147/prbm.S298948

24. Winter T, Riordan BC, Pakpour AH, Griffiths MD, Mason A, Poulgrain JW, et al. Evaluation of the English Version of the Fear of COVID-19 Scale and Its Relationship with Behavior Change and Political Beliefs. International journal of mental health and addiction. 2020; doi:10.1007/s11469-020-00342-9

25. Chia T, Oyeniran OI, Iorfa SK. Validation of the fear of the COVID-19 scale in Nigeria: Implications for public health practice. Journal of Taibah University Medical Sciences. 2021; doi:10.1016/j.jtumed.2021.05.006

26. Iversen MM, Norekvål TM, Oterhals K, Fadnes LT, Mæland S, Pakpour AH, et al. Psychometric Properties of the Norwegian Version of the Fear of COVID-19 Scale. International journal of mental health and addiction. 2021; doi:10.1007/s11469-020-00454-2

27. Mahmood QK, Jafree SR, Qureshi WA. The Psychometric Validation of FCV19S in Urdu and Socio-Demographic Association with Fear in the People of the Khyber Pakhtunkhwa (KPK) Province in Pakistan. International journal of mental health and addiction. 2020; doi:10.1007/s11469-020-00371-4

28. Barrios I, Ríos-González C, O'Higgins M, González-Urbieta I, García O, Almirón-Santacruz J, et al. Psychometric properties of the Spanish version of the Fear of COVID-19 scale in Paraguayan population. Irish journal of psychological medicine. 2021; doi:10.1017/ipm.2021.5

29. Huarcaya-Victoria J, Villarreal-Zegarra D, Podestà A, Luna-Cuadros MA. Psychometric Properties of a Spanish Version of the Fear of COVID-19 Scale in General Population of Lima, Peru. International journal of mental health and addiction. 2020; doi:10.1007/s11469-020-00354-5

30. Pilch I, Kurasz Z, Turska-Kawa A. Experiencing fear during the pandemic: validation of the fear of COVID-19 scale in Polish. PeerJ. 2021; doi:10.7717/peerj.11263

31. Magano J, Vidal DG, Sousa H, Dinis MAP, Leite Â. Validation and Psychometric Properties of the Portuguese Version of the Coronavirus Anxiety Scale (CAS) and Fear of COVID-19 Scale (FCV-19S) and Associations with Travel, Tourism and Hospitality. International journal of environmental research and public health. 2021; doi:10.3390/ijerph18020427

32. Stănculescu E. Fear of COVID-19 in Romania: Validation of the Romanian Version of the Fear of COVID-19 Scale Using Graded Response Model Analysis. International journal of mental health and addiction. 2021; doi:10.1007/s11469-020-00428-4

33. Reznik A, Gritsenko V, Konstantinov V, Khamenka N, Isralowitz R. COVID-19 Fear in Eastern Europe: Validation of the Fear of COVID-19 Scale. International journal of mental health and addiction. 2020; doi:10.1007/s11469-020-00283-3

34. Alyami M, Henning M, Krägeloh CU, Alyami H. Psychometric Evaluation of the Arabic Version of the Fear of COVID-19 Scale. International journal of mental health and addiction. 2020; doi:10.1007/s11469-020-00316-x

35. Pretorius TB, Padmanabhanunni A, Stiegler N, Bouchard JP. Validation of the fear of COVID-19 scale in South Africa: Three complementary analyses. Annales medico-psychologiques. 2021; doi:10.1016/j.amp.2021.10.010

36. Han JW, Park J, Lee H. Validity and Reliability of the Korean Version of the Fear of COVID-19 Scale. International journal of environmental research and public health. 2021; doi:10.3390/ijerph18147402

37. Chang KC, Hou WL, Pakpour AH, Lin CY, Griffiths MD. Psychometric Testing of Three COVID-19-Related Scales Among People with Mental Illness. International journal of mental health and addiction. 2020; doi:10.1007/s11469-020-00361-6

38. Satici B, Gocet-Tekin E, Deniz ME, Satici SA. Adaptation of the Fear of COVID-19 Scale: Its Association with Psychological Distress and Life Satisfaction in Turkey. International journal of mental health and addiction. 2020; doi:10.1007/s11469-020-00294-0

39. Perz CA, Lang BA, Harrington R. Validation of the Fear of COVID-19 Scale in a US College Sample. International journal of mental health and addiction. 2020; doi:10.1007/s11469-020-00356-3

40. Harper CA, Satchell LP, Fido D, Latzman RD. Functional Fear Predicts Public Health Compliance in the COVID-19 Pandemic. International journal of mental health and addiction. 2020; doi:10.1007/s11469-020-00281-5

41. Nguyen HT, Do BN, Pham KM, Kim GB, Dam HTB, Nguyen TT, et al. Fear of COVID-19 Scale-Associations of Its Scores with Health Literacy and Health-Related Behaviors among Medical Students. International journal of environmental research and public health. 2020; doi:10.3390/ijerph17114164
