# Supplementary material for: Parallel exploratory and confirmatory factor analysis of the Hungarian Fear of COVID-19 Scale in a large general population sample: a psychometric and dimensionality evaluation
Source: BMC Public Health. 2022 Jul 28;22:1438. doi: 10.1186/s12889-022-13789-3 (PMC9333073; doi:10.1186/s12889-022-13789-3)
Supplement: Supplementary file 2 — Additional file 2. [file 12889_2022_13789_MOESM2_ESM.docx]

| # Item | **FCV-19S Hungarian language questions** |
| --- | --- |
| 1. | Nagyon tartok a COVID-19-től. |
| 2. | Kellemetlen a COVID-19-re gondolnom. |
| 3. | A tenyerem nyirkossá válik, ha a COVID-19-re gondolok. |
| 4. | Félek, hogy meg fogok halni a COVID-19 miatt. |
| 5. | Idegességgel vagy szorongással tölt el, amikor a COVID-19-ről látok híreket vagy történeteket a közösségi média felületein. |
| 6. | Nem tudok aludni, mert aggódom, hogy elkapom a COVID-19-et. |
| 7. | A szívem gyorsabban vagy erősebben kezd dobogni, amikor arra gondolok, hogy elkaphatom a COVID-19-et. |
